# Effect of surgical mask on fMRI signals during task and rest

**DOI:** 10.1038/s42003-022-03908-6

**Published:** 2022-09-21

**Authors:** Benjamin Klugah-Brown, Yue Yu, Peng Hu, Elijah Agoalikum, Congcong Liu, Xiqin Liu, Xi Yang, Yixu Zeng, Xinqi Zhou, Xin Yu, Bart Rypma, Andrew M. Michael, Xiaobo Li, Benjamin Becker, Bharat Biswal

**Affiliations:** 1 The Clinical Hospital of Chengdu Brain Science Institute, MOE Key Laboratory for Neuroinformation, Center for Information in Medicine, School of Life Science and Technology, University of Electronic Science and Technology of China, No.2006, Xiyuan Avenue, West Hi-Tech Zone, Chengdu, Sichuan 611731 China; 2 Martimos Imaging Center, Harvard Medical School, Charlestown, MA 02129 USA; 3 The University of Texas at Dallas School of Behavioral and Brain Sciences, GR41800 W. Campbell Road, Richardson, TX 75080-302 USA; 4 Duke Institute for Brain Sciences, Duke University, Durham, NC USA; 5 Department of Biomedical Engineering, New Jersey Institute of Technology, Newark, NJ USA

**Keywords:** Neuroscience, Physiology

## Abstract

Wearing a face mask has become essential to contain the spread of COVID-19 and has become mandatory when collecting fMRI data at most research institutions. Here, we investigate the effects of wearing a surgical mask on fMRI data in *n* = 37 healthy participants. Activations during finger tapping, emotional face matching, working memory tasks, and rest were examined. Preliminary fMRI analyses show that despite the different mask states, resting-state signals and task activations were relatively similar. Resting-state functional connectivity showed negligible attenuation patterns in mask-on compared with mask-off. Task-based ROI analysis also demonstrated no significant difference between the two mask states under each contrast investigated. Notwithstanding the overall insignificant effects, these results indicate that wearing a face mask during fMRI has little to no significant effect on resting-state and task activations.

## Abstract (web summary)

Wearing a face mask during fMRI has little to no significant effect on resting-state and task activations.

## Introduction

The outbreak of COVID-19 in late 2019 was declared a global pandemic by the World Health Organization (WHO)^1^ in March 2020. The WHO issued a recommendation to wear face masks as one important precautionary measure to control transmission rates of the virus. Wearing a face mask significantly mitigates the risk of transmission and thus reduces the likelihood of person-to-person transmission^2^. The recommendation sparked heated debate in countries around the world. Concerns included that wearing the mask could be uncomfortable and inconvenient, interfering with activities of daily living, and questions about the effects of prolonged mask use^3,4^.

As MRI (and fMRI) imaging research facilities slowly resume scanning, most facilities are mandating the use of face masks in human subjects. Prior to the potential concern of wearing mask during MRI scanning, few studies have investigated the physiological and cognitive impact of wearing a face mask. Roberge and colleagues studied the underlying physiology under normal activity conditions (while wearing an N95 mask), such as walking at a slow pace^5,6^. Although the authors found a 3% increase in resistance during inhalation, signifying a higher demand for air/oxygen (O_2_) they concluded, that it did not significantly impact the underlying physiological processes. They suggested that comparatively lighter masks, such as standard surgical masks, might induce little to no differences in respiratory capacity and O_2_ saturation (the ratio of O_2_ saturated hemoglobin to the total hemoglobin in the blood). Although the above studies have focused on O_2_ differences, air demand and air composition are most often examined by measurements of end-tidal carbon dioxide (EtCO_2_), typically because both inspired O_2_ (in reduced volume) and expired lung air (with elevated CO_2_ accumulating during rebreathing) are mixed when a well-fitting mask is donned. However, while these studies suggest that physiological changes during facemask-wearing are minimal, potential effects on brain signals and activity are still not sufficiently investigated. Moreover, it is noteworthy that arterial blood would mainly be composed of increased CO_2_ (following a decreased O_2_) over time and may affect arterial vascular volume, especially in the gray matter, but more importantly, BOLD fMRI signal changes due to air composition are best acquired venously which is known to directly correspond to the blood oxygen levels (BOLD).

BOLD fMRI is predominantly used to study brain function. In both task challenge and the resting-state, the BOLD signal is dependent on the oxygen baseline levels and variations as well as conditions that affect oxygen consumption. One way to study cerebral oxygenation changes has been to use gas-air mixtures^7^. Another way has been to use breath-holding^8^. In breath-holding studies, participants are asked to hold their breath for a certain brief period, the task is expected to result in a reduction of oxygen and an accumulation of CO_2_ in the blood, particularly in the lungs, this introduces Hypercapnia^9,10^.

An initial and very recent study addressed the question of whether wearing a face mask can have an effect on the fMRI measurements. This recent work by Law et al. (2021) showed that wearing a face mask during fMRI induces higher levels of CO_2_ which potentially influence the BOLD signal, but minimally affects task activation^11^. This work is among the first to address the concern of possible effects of mask wearing on fMRI activation. However, the study conclusions were limited due to the comparably small sample size (*n* = 8) and the focus on a sensorimotor task, while the effects of wearing a face mask on frequently used cognitive, emotional, and resting state fMRI paradigms remain to be determined.

In this current study, based on a recent neuroimaging study showing negligible effect^11^ of wearing a mask, we thus sought to further examine fMRI data of mask states (masks-on and mask-off) for both resting-state and common task paradigms in a within-subject design in a larger sample of *n* = 35 healthy subjects.

## Results

### Participants and physiological measures

After subjecting the data to head motion exclusion criteria (translation < = 2 mm and rotation < = 2^o^) and subsequent removal of degraded images lead to a final sample of 35 right-handed healthy participants (15 males, 20 females, male mean age = 23.8 ± 1.13 years, female mean age = 23.4 ± 1.04 years) for all further analyses, Table 1. With respect SPO_2_ and HR, the paired *t*-test revealed that although mask-off was generally higher there was no significant difference for Pre-scan-O_2_ and Post-scan-O_2_ between mask states. Also, we found no differences between HR-pre-scan and HR-post-scan in both mask-on and mask-off. All results are summarized in Table 2.

**Table 1:** Demographic information of subjects.

	Number	Mean	SD	*P*-value (*t*-test)
Male	15			
Female	20			
Male age		23.8	1.13	
Female age		23.4	1.04	
STAI		32.08	8.20	a*
TAI		35.71	9.06	a*
BDI-ii		6.542	6.00	a*

*STAI* state-trait anxiety inventory, *TAI* test anxiety inventory, *BDI-II* Beck depression inventory-II, a* significant level less than 0.0001

**Table 2:** Saturation of peripheral oxygen (SpO_2_) and heart rate (HR)measured before and after scan for both mask-on and mask-off.

		Mask-on		Mask-off		*t*-value	*p*-value
	Pre-scan	mean	SD	mean	SD		
SPO_2_		97.74	1.46	98.07	1.55	−1.16	0.25
	Post-scan	98.48	1	98.51	1.12	−0.19	0.85
	Pre-scan	75.47	17	72.77	19.92	1.02	0.31
HR							
	Post-scan	69.81	19.22	71.94	14.52	−0.88	0.39
Pre + Post							
SPO_2_		98.11	1.09	98.29	1.11	−0.98	0.33
HR		72.64	16.64	72.29	15.81	0.15	0.88

### RS-fMRI signal

**Fig. 1:**
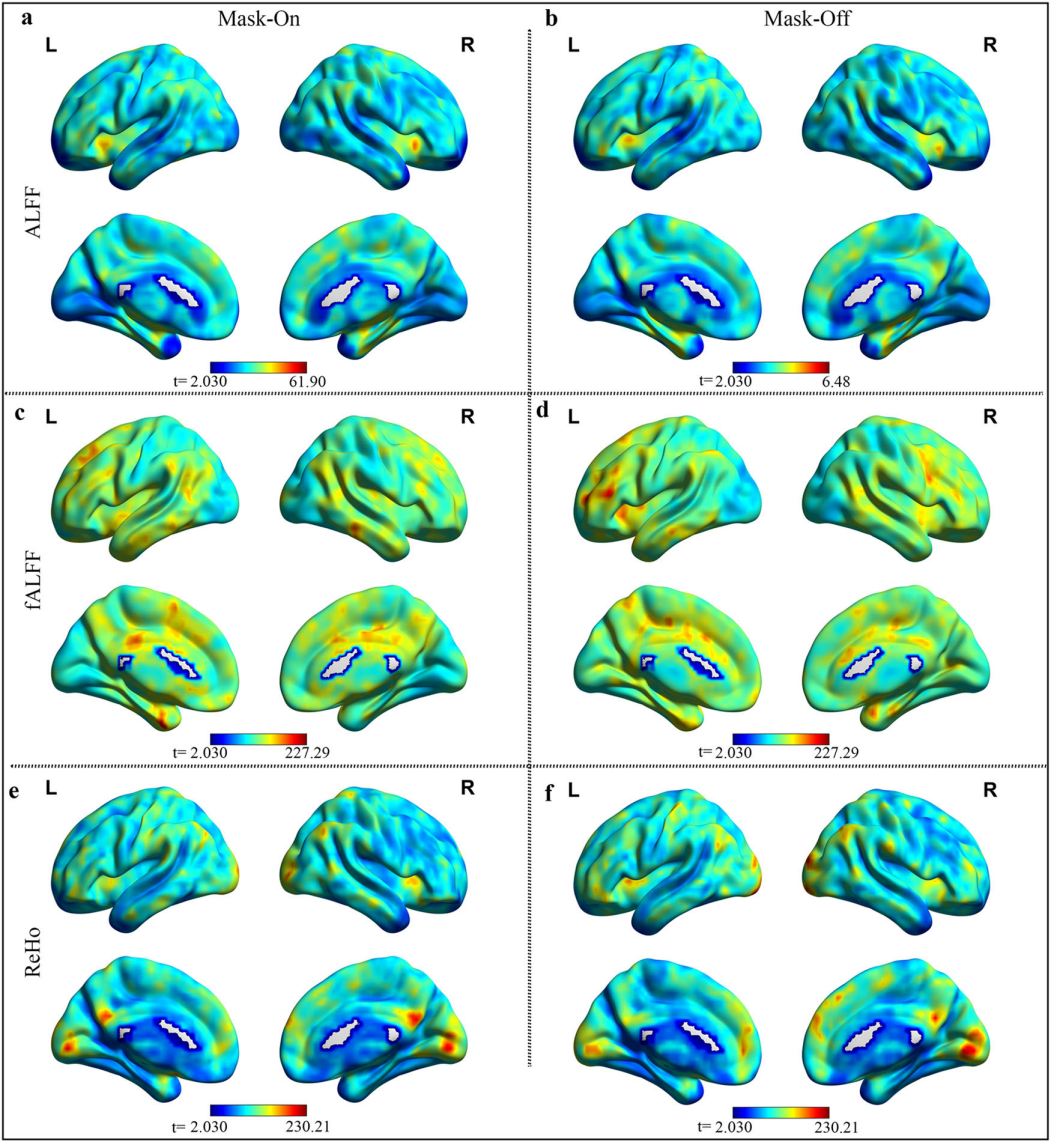
Mask-on and mask-off comparison of resting-state fMRI. (**a**, **b**), (**c**, **d**) and (**e**, **f**) represent one-sample *t*-test calculated for ALFF, fALFF, and ReHo, respectively in mask-on and mask-off states, the results are threshold at *p* < 0.05, FDR corrected.

Across the examined indices, we observe relatively similar ALFF and fALFF for both mask-on and mask-off. In both states, signals across a broad range of networks particularly in the default mode regions (PCC and the dLPFC) and salience networks (insula) Fig. 1a–d. The paired *t*-test between mask-on and mask-off demonstrated no significance (a threshold at *p* < 0.05, FDR corrected). With respect to the ReHo (Fig. 1e, f), we observe correlations in the default mode networks, and visual networks for both mask-on and mask-off, there was no significant difference between the two mask states, all uncorrected paired *t*-test maps are shown in Supplementary Fig. 1.

### Functional network connectivity

**Fig. 2:**
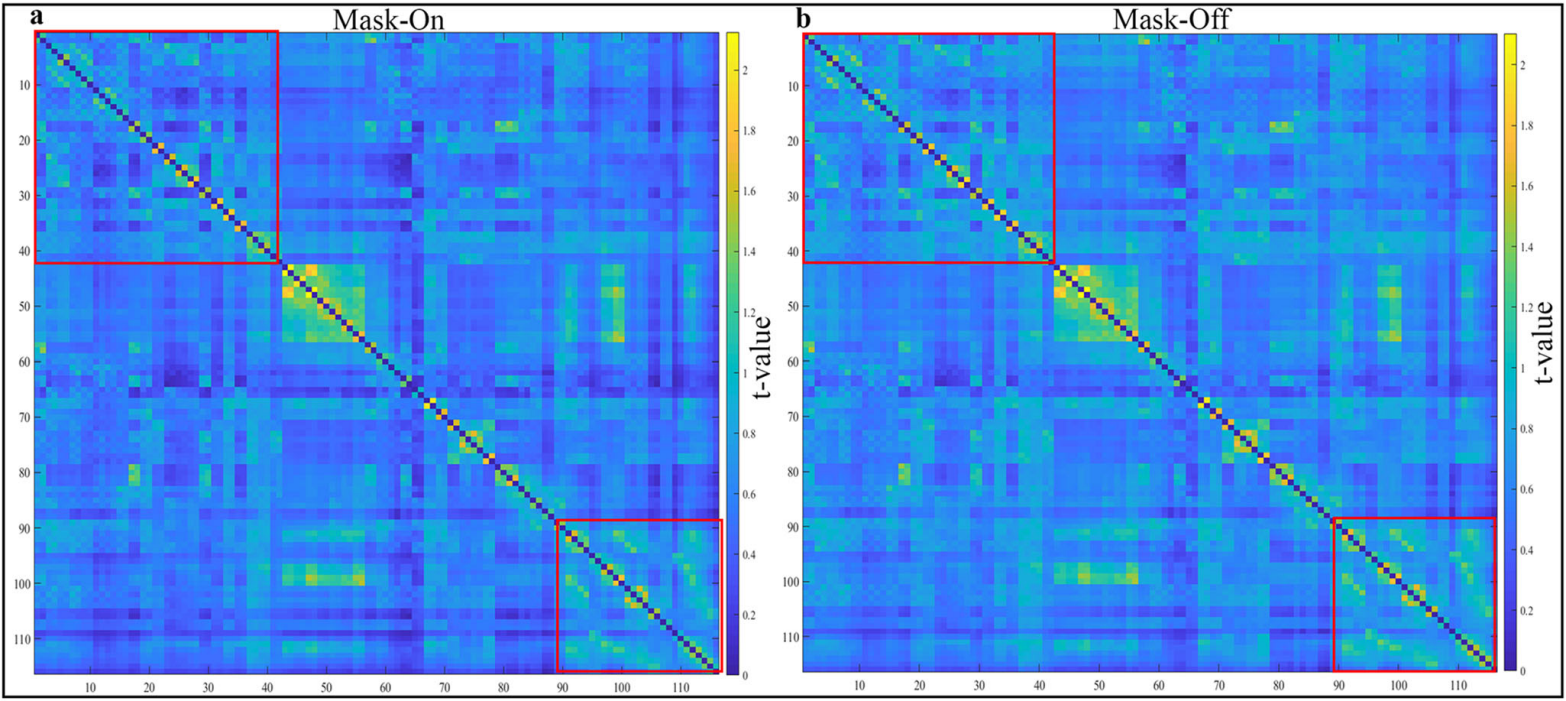
Statistical *t*-test of FC. **a**, **b** FC matrix of 116 ROIs extracted from the AAL template across all subjects for both mask state, color bar corresponds to the strength of *z*-values computed using one-sample *t* test (*p* < 0.05, FDR-corrected).

For the rsFC (Fig. 2a, b), the one-sample *t*-test computed within each mask state showed generally similar connectivity patterns across all the 116 ROIs. Although each state demonstrated both increased and increased within regional connectivity, the mask-off state was slightly higher compared to mask-on state. Paired *t*-test showed no significant difference between the two mask states (*p* < 0.05, FDR, corrected).

### Task analysis

#### Finger tapping task

**Fig. 3:**
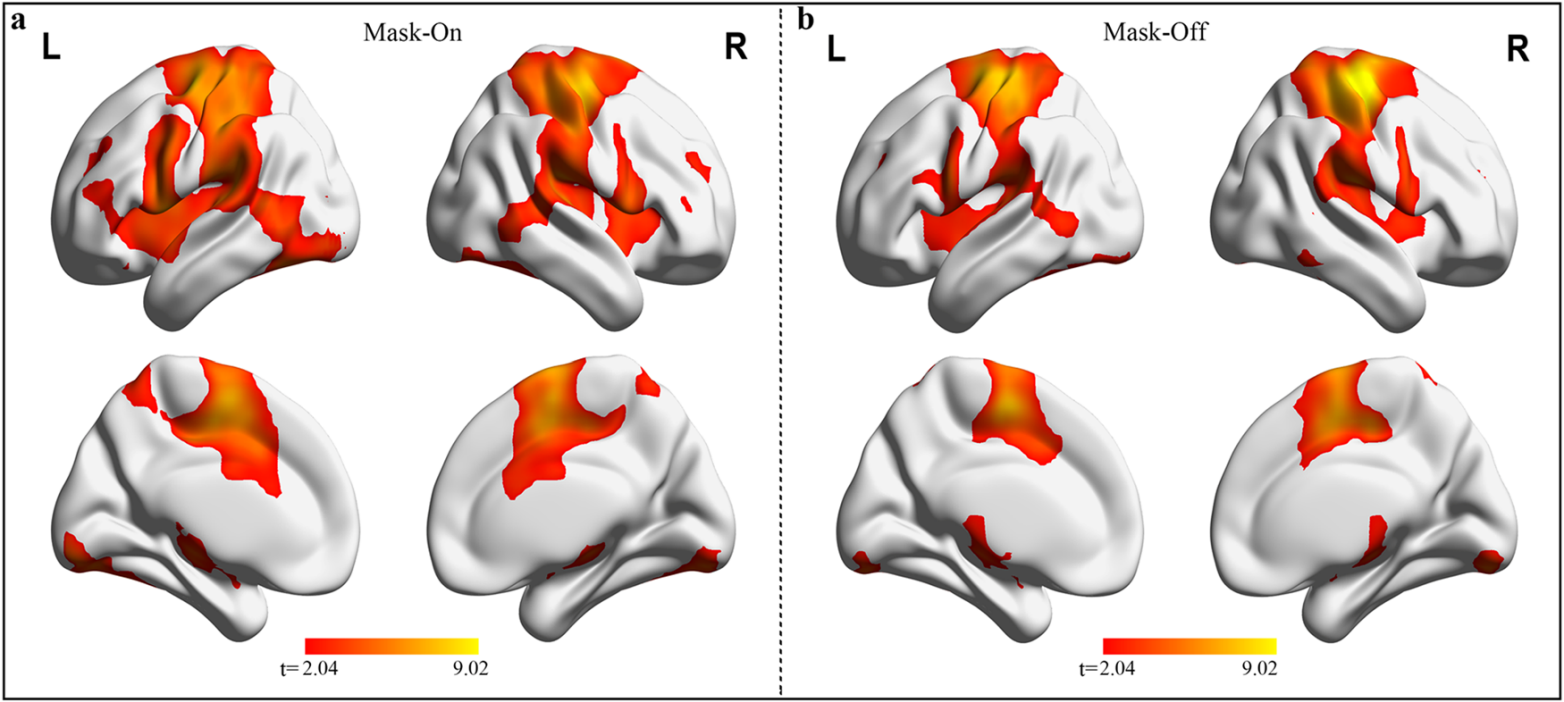
Finger tapping task-related activations (Finger tapping > fixation). **a** Regions associated with finger tapping task during mask-on state. **b** Activated regions during mask-off state. All regions shown were thresholded at *p* < 0.05, FDR-corrected, computed using one-sample *t*-test within each mask state. L left hemisphere, R right hemisphere, > greater than.

The whole-brain peak activation corresponding to the finger-tapping showed robust activations within each mask state. In both the mask-on and mask-off states, significant activations are located in the supplementary motor area (SMA) and postcentral gyrus, fusiform and calcarine of the visual networks, the cerebellum, the caudate, and some temporal regions, Fig. 3a, b. The difference between mask and no mask task activation was not significant (paired *t*-test, *p* < 0.05).

#### Emotional face-matching task

**Fig. 4:**
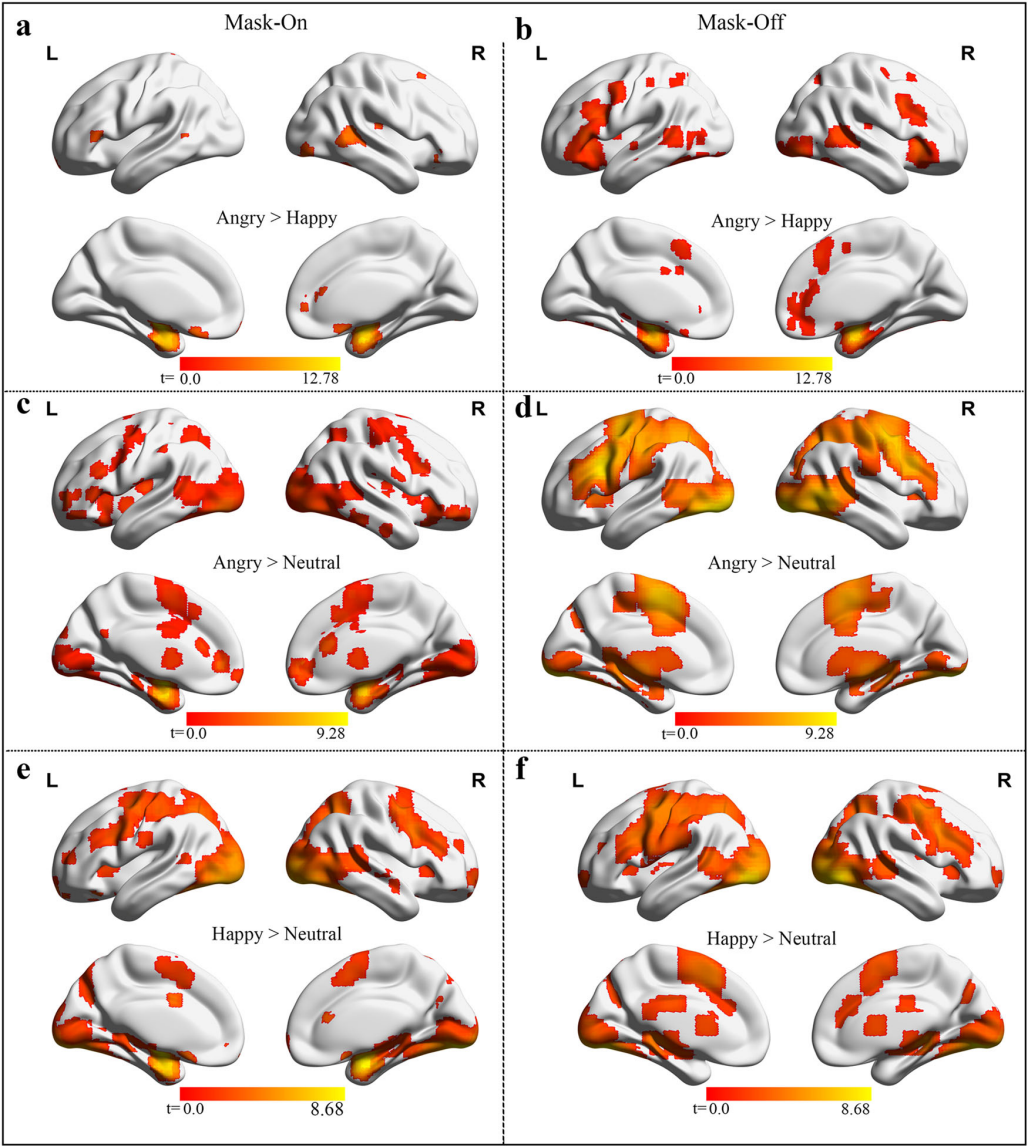
Emotional face matching associated activations in mask-on and mask-off. (**a**, **b:** angry faces > happy faces), (**c**, **d**: angry faces > neutral faces) and (**e**, **f**: happy faces > neutral faces) indicates the contrast computed for the emotional stimuli presented between the two mask states, respectively. The activations computed most occurs in the visual, hippocampus, middle/anterior cingulate, and prefrontal cortex (cluster-corrected threshold at *p* < 0.05). L left hemisphere, R right hemisphere, > greater than.

In this task, we initially determined the brain areas that showed the effect of attention and emotional processing between mask-on and mask-off. In the emotional task for both states, the activated regions are located in the thalamus, middle/anterior cingulate, prefrontal cortex, and hippocampus. Activations induced by angry contrasted with both happy and neutral pictures respectively were generally larger in cingulate and hippocampus during mask-off relative to mask-on state, Fig. 4a–d. Happy face contrast with the neutral face shared relatively similar regional activation generally across the motor, middle cingulate, and posterior cingulate gyrus, and hippocampus, Fig. 4e, f.

#### Working memory task

**Fig. 5:**
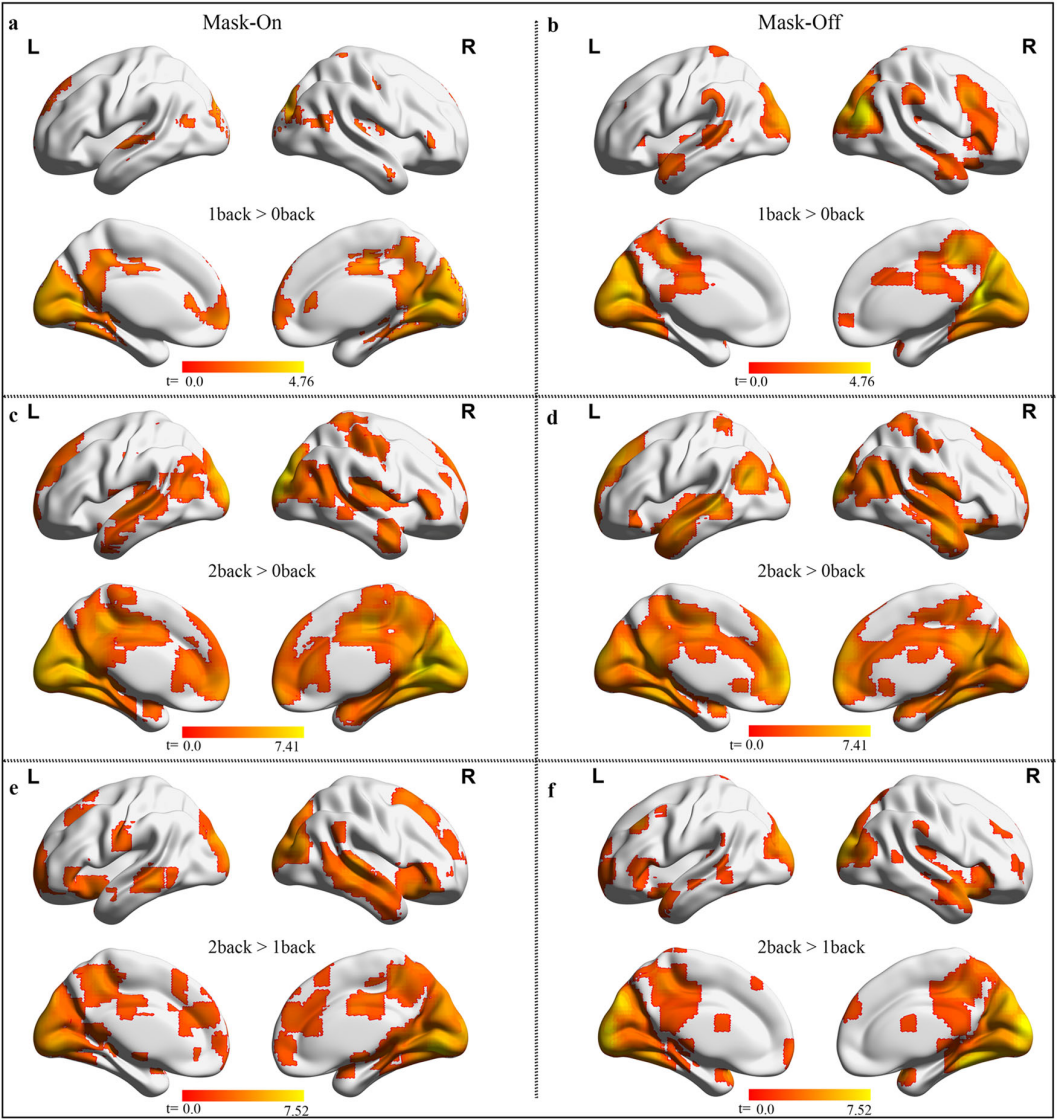
Working memory associated activations. (**a**, **b**: first memory load > baseline), (**c**, **d**: second memory load > baseline) and (**e**, **f**: second memory load > first memory load) show the activation pattern for each memory load contrast within each mask state. The pattern of activation is identical in both conditions (Activation cluster-corrected threshold of *p* < 0.05). L left hemisphere, R right hemisphere, > greater than.

This task includes three conditions with different working memory loads (0-back, 1-back, and 2-back). The 0-back was used as the baseline memory load. A similar pattern of activation occurred in the dorsolateral prefrontal cortex (dLPFC) for both mask-on and mask-off and each memory load. The regions activated are comparatively similar between mask-on compared to mask-off states, Fig. 5a–f. There was no significant activation difference between mask-on and mask-off states.

#### Task fMRI signal changes ROI analysis

**Fig. 6:**
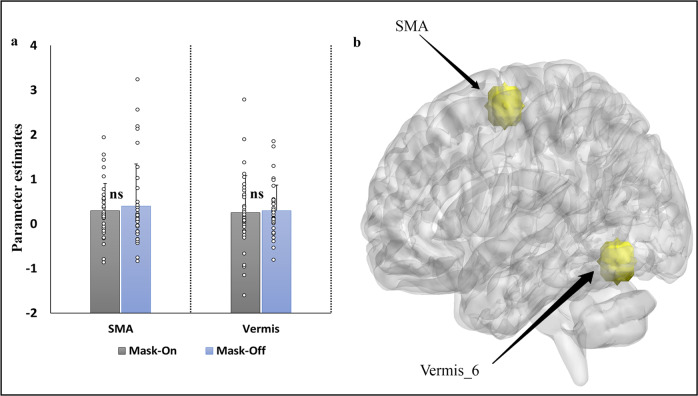
ROI analysis for finger tapping task. **a** shows the beta weights extracted for all subjects derived from the two ROIs in (**b**: SMA (*x* = 10, *y* = −4, *z* = 58) and vermis_6 (*x* = 6, −70, *z* = −16)) between the mask-on and mask-off. Differences between the two mask states computed at the ROIs was via paired *t*-test (*p* < 0.05). The error bars denote standard errors, ns not significant. L left hemisphere, R right hemisphere, > greater than, SMA supplementary motor area.

**Fig. 7:**
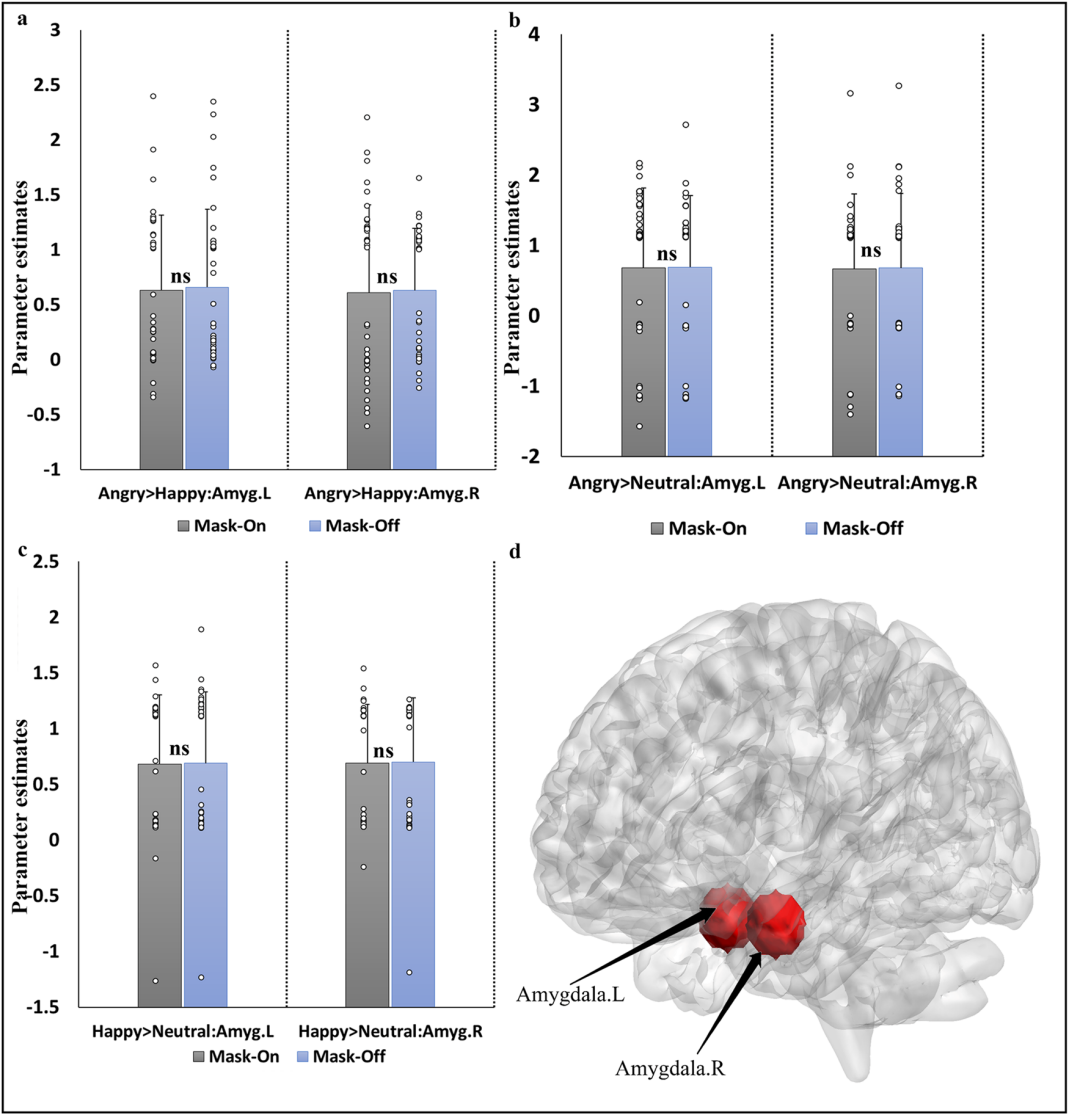
ROI analysis for emotional face-matching tasks. (**a**: angry face > happy faces, **b**: angry faces > neutral faces and **c**: happy face > neutral faces) shows the beta weights extracted for all subjects derived from the two ROIs in (**d**: left amygdala (*x* = −24, *y* = −2, *z* = −32) and right amygdala (*x* = 26, *y* = −6, *z* = −28) between the mask-on and mask-off states. The difference between the two conditions computed at the ROIs was via paired *t*-test (*p* < 0.05, Bonferroni corrected). The error bars denote standard errors, ns not significant. L left hemisphere, R right hemisphere, > greater than, Amyg amygdala.

**Fig. 8:**
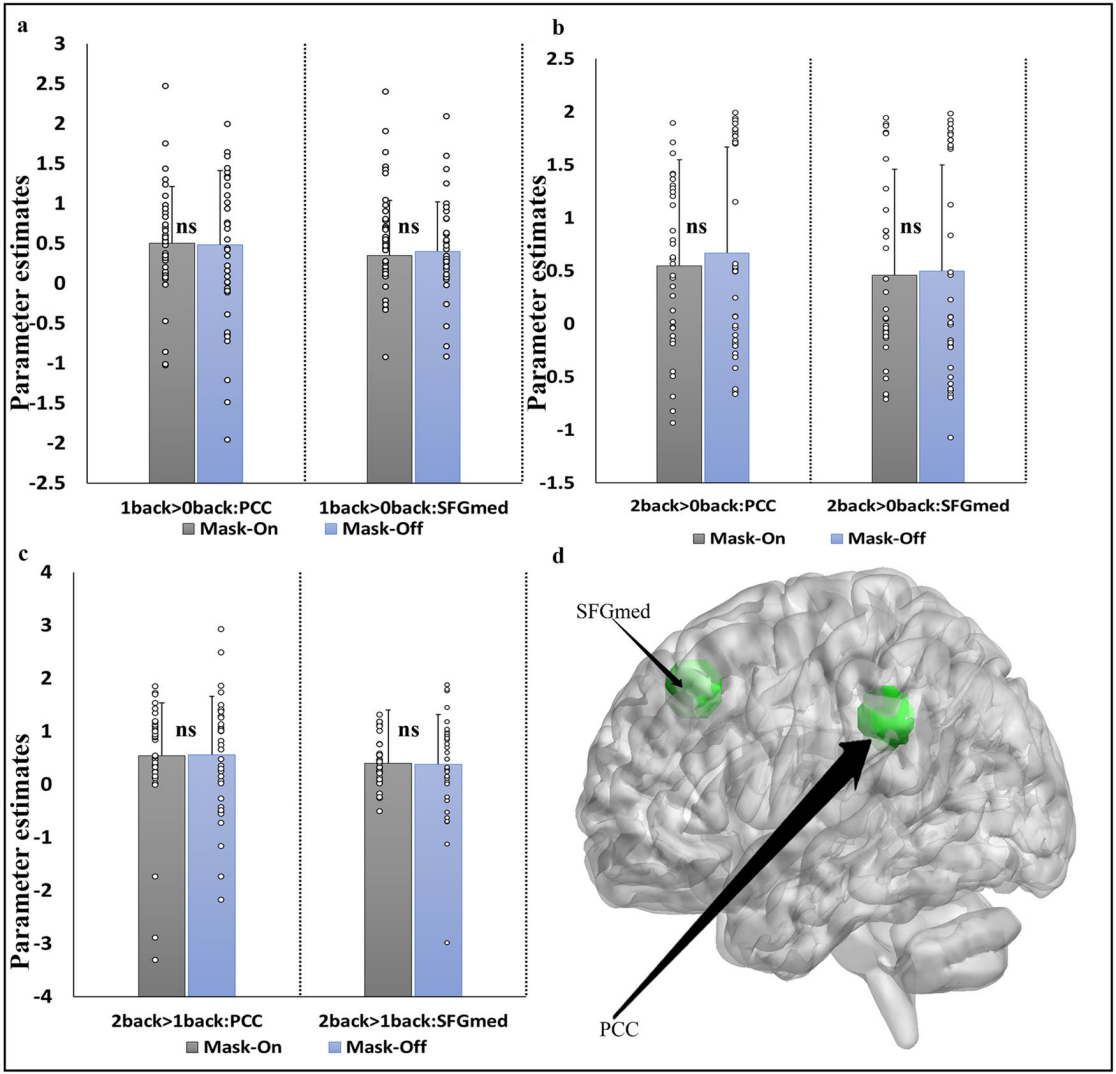
ROI analysis for working memory tasks. (**a**: angry faces > happy faces, **b**: angry faces > neutral faces and **c**: happy faces > neutral faces) shows the beta weights extracted for all subjects derived from the two ROIs in (**d**: superior frontal gyrus, medial (*x* = 2, *y* = 54, *z* = 20) and posterior cingulate cortex (PCC; *x* = 2, −44, *z* = −28)). The difference between the two conditions was examined via paired *t*-test (*p* < 0.05, Bonferroni corrected). Error bar shows standard error, ns not significant, L left hemisphere, R right hemisphere, > greater than.

ROI analysis was performed for the three independent tasks (ROIs see Methods). Paired *t*-test (threshold *p* < 0.05) were computed on the extracted parameter estimate values for the ROIs in the mask-on and mask-off states. The finger-tapping task showed no significant differences between mask-on and mask-off in the Vermis_6 and the SMA respectively (Fig. 6a, b). For the emotional face matching experiment, there was no significant difference between the two mask states for any of the contrasts examined (angry > happy, angry > neutral and happy > neutral; Fig. 7a–c) in the amygdala (Fig. 7d). Similarly, in the working memory task, there was no a significant difference in 1-back > 0-back and 2-back > 1-back contrast obtained from the ROIs (Fig. 8a–c) of the two regions (Fig. 8d).

## Discussion

We employed resting-state and task-based approaches to examine whether wearing a surgical mask could impact fMRI signals during scanning. Generally, we observed consistent resting-state signals and task activation between the two mask states. (1) there were no significant rsFC differences between mask-on and mask-off on the whole-brain level (2) the overall finding in the task experiments showed a relatively similar activation pattern in both mask-on and mask-off states with no significant differences in activations. Our results, therefore, confirmed the lack of effects of mask wearing on activation patterns observed in^11^ and extend the findings to additional cognitive and emotional tasks as well as resting-state fMRI.

The rs-signal analysis demonstrated relatively similar ALFF, fALFF, and connectivity fluctuations in ReHo. Mask-off demonstrated insignificant differences in all the three measures compared to mask-on, statistical differences (uncorrected, Supplementary Fig. 1) suggesting that there exists a subtle variation in air mixture (maybe via CO_2_ accumulation, during mask-on as also described in^11^) resulting in slight activation change between the two mask states but the effect is insignificant and does not reflect any underlying signals differences during rs-fMRI.

The rsFC showed no significant differences between the two mask states, however, the overall connectivity pattern demonstrated were comparable yet, the mask-on exhibited relatively insignificant lower connectivity strength compared to mask-off, this may be due to increasing CO_2_ in the air mixture. In general, both the three resting state measures and FC, showed mask-on being slightly lower compared to mask-off. This observation may be the result of increased cerebral perfusion causing a decrease in arterial SPO_2_ as opposed to the net increase in venous O_2_.

For task analysis, the finger tapping task showed similar patterns in both mask-on and mask-off, particularly in SMA, basal ganglia, and cerebellar regions. Earlier studies have revealed that SMA was implicated in various forms of motor activity such as basic voluntary movement and bimanual coordination^12,13^. Also, sequential and repetitive movement has been linked to the activation of the basal ganglia^14,15^. Thus, we expected these activation patterns to be evident under both mask-on and mask-off states. Our result was consistent with^11^ in which they also found no activations difference in the sensory-motor task.

Examination of the emotional face-matching task employing neutral face as the baseline revealed that angry faces engaged the prefrontal cortex for both conditions with differences occurring in the middle cingulate gyrus, the thalamus, visual cortex, hippocampus, and the dLPFC. Furthermore, in both angry and happy faces conditions, identical activations appeared under each mask state relative to the neutral face condition. For the ROI analysis, BOLD signals in the mask-on condition were lower relative to the mask-off condition for all the various contrasts calculated.

The working memory task showed robust activations in regions such as the dLPFC, visual cortex, SMA middle/anterior cingulate, the thalamus, and the PCC, which were expected under the two mask-off states. Similar patterns of activation were shown to imply the corresponding cognitive and behavioral responses in the experiments. Activations between the two mask states are generally similar across the brain, indicating that there was no influence of mask on the general task activations responses across the memory loads. This also suggests a similar neural activity for each memory load and is consistent with studies investigating the maintenance of working memory in humans^16^. Although these activation patterns in both mask states are not affected some studies have shown a similar effect under hypoxia conditions^17^. Working memory performance reflects more response to BOLD signal which corresponds to a greater task proficiency, that is, increasing task difficulty will produce a greater response in the brain. The PCC was implicated in higher memory load^18^ consistent with our results. However, further investigation would be needed to clarify whether the effects were due to some underlying cognitive process.

It is noteworthy to mention that the findings must be considered in the context of limitations. Firstly, while we were able to measure the SPO_2_ pre- and post-scan this might not reflect the true BOLD fMRI signal changes compared to if EtCO2 were to be measured and airflow was controlled in-scanner as done in^11,19^. Secondly, pre-scan wearing of the mask was short during the experimental session, we also modified the experiment to include a shorter task duration to allow for minimal and convenient task performance for both mask-on and mask-off states, longer duration of wearing of mask pre- and post-scan will therefore improve task responses. Future studies need to examine the effects of longer mask wearing as well as conducting the task separately. Also, the reduction in peripheral SPO_2_ as an estimate of saturations of arterial oxygen– SaO_2_, may correspond to changes in gray matter and remain speculative

Overall, our results demonstrated across all measures including resting-state and task activations both mask-on and mask-off states exhibited similar patterns. This may reflect the negligible to no effect of wearing a surgical mask on fMRI signal. In essence, these results confirm and complement the results in^11^ by extending the data size and the useful experimentation that reflects the most used paradigms in fMRI.

## Methods

### Subjects

Thirty-seven right-handed, healthy young adults (Mean age = 23.6; range = 21–27) were required to wear a surgical face mask in one of two fMRI acquisition sessions. All the participants were recruited from a campus setting and were all full-time undergraduate or graduate-level students with at least 14 years of educational training and were considered to have a commendable IQ level. All participants underwent a thorough examination of vital capacity (level of respiratory muscle weakness, lung volume, and breathing rate) before scanning in the University of electronic science and technology of China (UESTC) hospital before the experiment. To exclude subjects with high levels of depression or anxiety at pathological-relevant levels, participants were screened by means of two scales (Chinese Beck Depression Inventory, C-BDI^20^; State-Trait Anxiety Inventory, STAI^21^).

Before scanning, all subjects practiced the tasks. All study procedures were approved by the local Ethics Committee of UESTC. All subjects provided written consent to participate in this study. Part of the consent included the exact information about the scanning procedure and psychological assessment. The study was approved by the Ethics Committee of the clinical hospital of Chengdu Brain Science Institute (CBSI) and adhered to the latest revision of the Declaration of Helsinki.

### Data acquisition

MRI data were collected using an MRI scanner (3.0 T, Discovery MR750, GE, USA). The functional images were collected using a gradient-echo echo-planar imaging sequence. The scanning parameters were as follows: TR/TE = 2000 ms/30 ms; field of view = 240 × 240 mm^2^; flip angle = 90°; matrix size = 64 × 64 and thickness = 4 mm. Axial slice number = 42 with slice thickness = 3 mm and gap = 0. Data were collected for both resting and task fMRI for both mask-on and mask-off states. The resting-state fMRI data were acquired in an 8 min single run containing 240 image volumes. The first five volumes of the resting scan were discarded to ensure steady-state longitudinal magnetization. During the resting-state scan, participants were instructed simply to keep their eyes closed and not to think of anything in particular.

### Physiological and psychological tests

All physiological and psychological tests were performed prior to the MRI scan sessions. The initial steps involve asking participants to rest for 5 min to lower the heart rate. The scanning section included two assessments during which participants were randomly assigned to wearing a mask (mask-on) and not wearing a mask (mask-off). That is subjects were scanned twice, if a subject enters the scanner with mask-on, he/she will be scanned again without mask, and the SPO_2_ saturations measured accordingly. Prior to the participant donning a mask, we removed the metal strip from the upper part of the face mask. To reduce the effect of removing the metal strips, we pasted double-faced adhesive tape to each mask where we removed metal strips to maintain gas tightness. For the mask-on condition, the participants completed the following psychological tests: State-Trait Anxiety Inventory, Test Anxiety Inventory, and Beck Depression Inventory-II. Participants also practiced the three tasks without taking off the mask. After the psychological tests, a pulse oximeter was placed on the index finger for 3 min to measure SPO_2_ saturation and heart rate (henceforth referred to as pre-scan-O_2_). The participants then entered the scanner. After the MRI scans were completed, O_2_ saturation and heart rate were measured again (henceforth referred to as Post-scan-O_2_). The results of the physiological measurements were compared within each mask state between before and after the MRI session using *t*-test, statistical significance was thresholded at *p* < 0.05. A *t*-test was used to test the difference within mask state, and a paired *t*-test was used between mask-on and mask-off, statistical significance was thresholded at *p* < 0.05.

### Preprocessing

All the images were preprocessed using DPARSF1 (http://rfmri.org/DPARSF) applying the following steps: the image acquisition between slices was time corrected, images were realigned to remove possible movement artifacts, images were normalized to a standard EPI template, each subject image was warped into a standard space with a resolution of 3 × 3 × 3 mm using the Montreal Neurological Institute (MNI) template, and finally the images were smoothed with an 8 mm full width at half maximum (FWHM) Gaussian kernel. We did not regress global signal as this was assumed to introduce a negative correlation and to diminish the actual signal being analyzed. The head movement parameters were estimated in the three directions (*x*, *y*, *z*) along with the angular rotation on each axis (pitch, roll, and yaw) using the Friston 24-parameter model^22^. We set the motion threshold at translation < 2 mm and rotation < 2° to avoid extreme head motion, subjects with values more than the thresholds were excluded from further analysis. The framewise displacement (FD)^23^ was evaluated for each subject using the formula;

1 $${FD}=\frac{1}{t-1}\mathop{\sum }\limits_{i=2}^{t}\sqrt{{{\left | \triangle {d}_{{x}_{i}^{1}} \right | }}^{2}+{{\left | \triangle {d}_{{y}_{i}^{1}} \right | }}^{2}+{{\left | \triangle {d}_{{z}_{i}^{1}} \right | }}^{2}+{{\left | \triangle {d}_{{x}_{i}^{2}} \right | }}^{2}+{{\left | \triangle {d}_{{y}_{i}^{2}} \right | }}^{2}+{{\left | \triangle {d}_{{z}_{i}^{2}} \right | }}^{2}}$$

where t is the number of timepoint in the fMRI; ${x}_{i}^{1}/{x}_{i}^{2}$,${y}_{i}^{1}/{y}_{i}^{2}$ and ${z}_{i}^{1}/{z}_{i}^{2}$ are translation and rotation at *t*^*th*^ timepoint in all three directions (*x, y*, and *z*) and $\triangle {d}_{{x}_{i}^{1}}={x}_{i}^{1}-{x}_{i-1}^{1}$(*Eq. 2*). We set the mean displacement threshold as FD < = 0.5.

### Data analysis

All data analysis was done using spm12 software (https://www.fil.ion.ucl.ac.uk/spm/software/spm12/) as well as spm12-dependent scripts for batch computations.

### RS-fMRI signal

Resting-state signal measures including the amplitude of low-frequency fluctuations (ALFF), fractional ALFF (fALFF), and regional homogeneity (ReHo) were computed for all subjects in both conditions. The voxel-wise ALFF measure is the mean square root of the power in the chosen frequency windows. The fALFF specifies the relative contribution of oscillations in a selected low-frequency range to the signal differences over the whole frequency range specified. In this calculation, the similarity of the time series of a given voxel was measured to those of its 27 nearest neighbors in a voxel-wise way and then Kendall’s coefficient of concordance (KCC) value was given to each center voxel. All ALFF images were estimated by extracting power spectra through Fast Fourier Transform and computing the sum of amplitudes within a low-frequency band (0.01–0.08 Hz). The fALFF was also computed as the ratio of the amplitude within the same frequency range as the ALFF to the total amplitude over the full frequency range of 0.01–0.1 Hz. We computed the ReHo using KCC. The ReHo map was spatially smoothed with a 6 × 6 × 6 mm FWHM Gaussian kernel.

### Functional connectivity

We computed the FC via DPARSF using the AAL whole brain atlas^24^ comprising 116 ROIs. Averaged time courses were obtained from all 116 ROIs, and voxel-wise correlation analysis was performed to create the FC map. The correlation coefficient map was then converted to a z map by Fisher’s r-to-z transformation to improve normality. We further extracted the correlation matrices for each mask state and performed paired tests between the mask-on and mask-off.

### Task study design and analysis

The preprocessing steps were the same as for the resting state data. We conducted three different tasks for mask-on and mask-off conditions including finger tapping, emotional facial matching task, and working memory. These experiments represent the basic motor, cognitive, and emotional processes that can be used to evaluate the effect of the two conditions. For each subject, a first-level statistical analysis was conducted by applying a high pass filter set at 128 s, each mask state (mask-on and mask-off) was then modeled using the canonical hemodynamic function of the block designs in the experiments. To detect within-condition activations, second-level statistical computations were performed in each of the tasks. Contrasts were computed within each condition for all tasks.

Although some studies have suggested that exposure to a relatively low O_2_ level similar to mild hypoxia might not affect overall cognition and some important motor performance^25,26^, there have also been reports indicating that mild hypoxia might affect complex cognitive ability including memory, attention, and a task involving perceptual-motor performance^27,28^, based on these reports, we chose the three widely use fMRI task namely, finger tapping^29,30^, emotional face-matching^31^ and n-back working memory^32^. We computed the paired *t*-test (*p* < 0.05) between the two conditions.

### Finger tapping task

We employed a finger tapping task in the current study due to its simplicity and ability to measure subtle cortical motor integrity and provide corresponding motor activations that can be observed using fMRI. Also, O_2_ modulations in response to BOLD fluctuation are well preserved during motor action^33,34^, allowing for estimation of neuronal activity under a reduced level of O_2_. In this task, a cross and a sentence were presented alternately. When the task started, a fixation was presented and lasted 34 s, then an instruction that was also the task sentence (‘please move your finger’) was presented and lasted 24 s, and this pattern was repeated. Except for the first fixation, the rest of the fixations, as well as instruction, lasted 24 s. The total task time was 226 s.

### Emotional face matching task

In this experiment, we used a block-design fMRI paradigm which has been shown to determine responses to angry faces^35,36^ in emotionally related regions such as the amygdala. We modified the task to consist of 2 runs and each run comprised 6 blocks of facial stimuli as well as 2 blocks of non-facial stimuli. We utilized the Asian facial stimuli obtained from the Asian facial expression database^37^. During the face-processing blocks, a trio of condition-specific (happy, angry, or neutral expressions) facial stimuli were presented on the screen. Participants were asked to select from 2 pictures (bottom) and identify which one was identical to the target picture (top), then a keypress reaction was performed to record the results. Each block comprised 4 condition-specific trials, balanced for gender. During the non-facial blocks, a trio of simple geometric shapes (circles and ellipses) was presented on the screen. Similarly, participants were required to select one of the two shapes (bottom) that matched the target shape (top). All blocks started with a brief instruction (“face match” or “shapes match”) that lasted 2 s. Within each block, each trial was presented for 4 s with an interstimulus interval (ISI) of 1–3 s (mean, 2 s). The total task time was 336 s.

### Working memory task

Participants performed an n-back task^38^ consisting of letters that required the maintenance and continual update of relevant working memory information. The n-back task had two different levels of complexity: 1-back and 2-back tasks aimed at sustaining loads and mental manipulation. Participants also performed a control task (0-back) in which they were asked to identify a prespecified letter (i.e., an “X”). During 1-back task, participants were required to identify whether the letter appeared on the screen (the “target” stimulus) matched the letter previously presented on the screen (the “cue” stimulus). Similarly, during 2-back task, participants were asked to compare whether the letter that appeared on the screen (the “target” stimulus) matched the one before the previously presented letter on the screen (the “cue” stimulus). Participants were asked to respond by pressing buttons 2 or 3 if the target was identical or different from the cue, respectively. To reduce visual and phonological strategies, we used phonologically closed letters with upper and lower case. Thus, the following characters were presented: b, B, d, D, g, G, p, P, t, T, v, V. Participants were told to ignore the case of the letters. Letters were presented for 500 ms with a fixed interstimulus interval of 1500 ms. There were 4 runs, each run had 3 blocks (0-1-or 2 block), and each block had 36 trials including 12 targets. Prior to each task block, an instruction screen (0, 1-, or 2-back) was presented for 2000 ms. A 4000 ms blank screen separated the instructions from the onset of the first letter. Task blocks were separated by an 8000 ms fixation cross. The total task time was 270 s.

### ROI-based analysis for all task fMRI

To allow a more sensitive analysis we additionally included a regions-of-interest (ROIs) analysis. ROIs were created (radius 6 mm) using MarsBar toolbox^39^ implemented in SPM. We defined anatomical masks for each of the tasks: finger tapping (SMA; *x* = 10, *y* = −4, *z* = 58 and vermis; *x* = 6, *y* = −70, *z* = −16), emotional face matching (left amygdala; *x* = −24, *y* = −2, *z* = −32 and right amygdala; *x* = 26, *y* = −6, *z* = −28) and working memory (superior frontal gyrus, medial; *x* = 2, *y* = 54, *z* = 20 and posterior cingulate cortex; *x* = 2, *y* = −44, *z* = 28). These ROIs were based on the meta-analytic brain regions derived from the Neurosynth database (https://neurosynth.org/). All ROI labeling was based on Automated Anatomical Labeling (AAL) atlas^24^ in MNI space. Prior to the analysis, a multiple regression was computed to obtain activation maps, and then ROIs were used to obtain parameter estimate values. Paired *t*-test was used to evaluate the difference between the values of the two conditions (significant threshold set at *p* < 0.05).

### Statistics and reproducibility

To ensure statistical simplicity and reproducibility of our results, we adopted a two-step approach: We randomly determined the mask state (mask on and mask off) of each subject pre and post scanning. The data collected from each subject were first grouped into the pre- and post-scanning, results for each mask condition were later combined for all *n* = 35 subjects. We compared the t-maps resulting from both the resting state experiment and the task experiment, each consisting of a one-sample *t*-test for the within mask state effect and a paired *t*-test for between mask state effect in the whole brain (excluding voxels outside the brain).

### Reporting summary

Further information on research design is available in the Nature Research Reporting Summary linked to this article.

## Supplementary information

Supplementary Information

Description of Additional Supplementary Files

Supplementary Data 1

Reporting Summary

## Data Availability

All the data was obtained from the University of electronic science and technology of China. The data is available from the corresponding authors upon acceptance of this manuscript and a reasonable request. All data points used to generate statistical plots of the ROIs analysis in Figs. 6, 7 and 8 are provided in Supplementary Data 1.
