# Supplementary material for: Effect of surgical mask on fMRI signals during task and rest
Source: Commun Biol. 2022 Sep 21;5:1004. doi: 10.1038/s42003-022-03908-6 (PMC9491667; doi:10.1038/s42003-022-03908-6)
Supplement: Supplementary file 1 — Supplementary Information [file 42003_2022_3908_MOESM1_ESM.pdf]

Effect of surgical Mask on fMRI signals during task and rest

Supplementary Figure

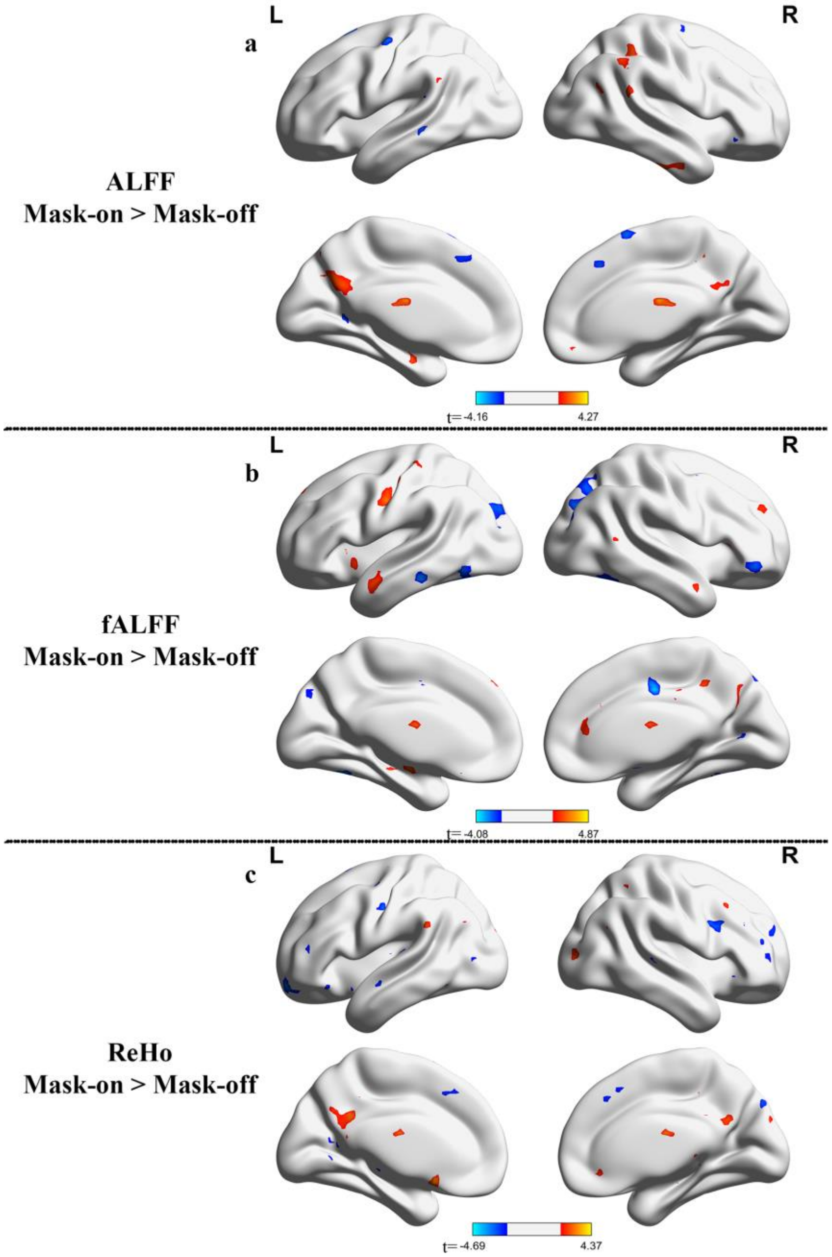

Fig. 1 uncorrected ($p<0.05$) difference between mask-on and mask-off states
