# Supplementary material for: Effect of surgical mask on fMRI signals during task and rest
Source: Commun Biol. 2022 Sep 21;5:1004. doi: 10.1038/s42003-022-03908-6 (PMC9491667; doi:10.1038/s42003-022-03908-6)
Supplement: Supplementary file 2 — Description of Additional Supplementary Files [file 42003_2022_3908_MOESM2_ESM.docx]

**Description of Additional Supplementary Files**

**File name:** Supplementary Data 1

**Description:** The source data behind the graphs in figures 6-8
