# Supplementary material for: Effect of surgical mask on fMRI signals during task and rest
Source: Commun Biol. 2022 Sep 21;5:1004. doi: 10.1038/s42003-022-03908-6 (PMC9491667; doi:10.1038/s42003-022-03908-6)
Supplement: Supplementary file 4 — Reporting Summary [file 42003_2022_3908_MOESM4_ESM.pdf]

nature portfolio

Corresponding author(s): Benjamin Klugah-Brown

Last updated by author(s): Aug 29, 2022

Reporting Summary

Nature Portfolio wishes to improve the reproducibility of the work that we publish. This form provides structure for consistency and transparency in reporting. For further information on Nature Portfolio policies, see our Editorial Policies and the Editorial Policy Checklist.

Statistics

For all statistical analyses, confirm that the following items are present in the figure legend, table legend, main text, or Methods section.

n/a	Confirmed	
☐	☒	The exact sample size (*n*) for each experimental group/condition, given as a discrete number and unit of measurement
☒	☐	A statement on whether measurements were taken from distinct samples or whether the same sample was measured repeatedly
☐	☒	The statistical test(s) used AND whether they are one- or two-sided<br>*Only common tests should be described solely by name; describe more complex techniques in the Methods section.*
☐	☒	A description of all covariates tested
☐	☒	A description of any assumptions or corrections, such as tests of normality and adjustment for multiple comparisons
☒	☐	A full description of the statistical parameters including central tendency (e.g. means) or other basic estimates (e.g. regression coefficient) AND variation (e.g. standard deviation) or associated estimates of uncertainty (e.g. confidence intervals)
☒	☐	For null hypothesis testing, the test statistic (e.g. *F*, *t*, *r*) with confidence intervals, effect sizes, degrees of freedom and *P* value noted<br>*Give P values as exact values whenever suitable.*
☒	☐	For Bayesian analysis, information on the choice of priors and Markov chain Monte Carlo settings
☒	☐	For hierarchical and complex designs, identification of the appropriate level for tests and full reporting of outcomes
☒	☐	Estimates of effect sizes (e.g. Cohen's *d*, Pearson's *r*), indicating how they were calculated

Our web collection on statistics for biologists contains articles on many of the points above.

Software and code

Policy information about availability of computer code

Data collection	No software was used
Data analysis	Preprocessing. All the images were preprocessed using DPARSF1 (http://rfmri.org/DPARSF). All data analysis was done using spm12 software (https://www.fil.ion.ucl.ac.uk/spm/software/spm12/)

For manuscripts utilizing custom algorithms or software that are central to the research but not yet described in published literature, software must be made available to editors and reviewers. We strongly encourage code deposition in a community repository (e.g. GitHub). See the Nature Portfolio guidelines for submitting code & software for further information.

Data

Policy information about availability of data

All manuscripts must include a data availability statement. This statement should provide the following information, where applicable:

- Accession codes, unique identifiers, or web links for publicly available datasets
- A description of any restrictions on data availability
- For clinical datasets or third party data, please ensure that the statement adheres to our policy

All the data was obtained from the University of electronic science and technology of China.
The data is available from the corresponding authors upon acceptance of this manuscript and a reasonable request

Human research participants

Policy information about studies involving human research participants and Sex and Gender in Research.

Reporting on sex and gender	In this current studies we collected data from both male a female participants, Although we consider the demographic age difference between male and females, we didn't compute gender differences as these participants were all healthy with similar clinical traits. Also, our main focus was to examine the general effect in these healthy participant.
Population characteristics	All subjects were right-handed, healthy young adults (Mean age = 23.6; range = 21-27) . All the participants were recruited from a campus setting and were all full-time undergraduate or graduate-level students with at least 14 years of educational training, and were considered to have a commendable IQ level. All participants underwent a thorough examination of vital capacity (level of respiratory muscle weakness, lung volume, and breathing rate) before scanning in the University of electronic science and technology of China (UESTC) hospital before the experiment. Gender was balanced to ensure against possible gender effect in the experiment.
Recruitment	Participants were recruited via campus group platforms and flayer distribution on campus. We outlined the procedures of the tasks on the flayers and in the group platforms, e.g. participants will be required to wear surgical mask through the entire experimental period. Prior to the final selections, participant were sent to campus hospital to examine legibility , participants who were not fit were excluded. This ensured against self selection, which might bias the results such as participants not completing experiments due to high level of anxiety and Claustrophobia
Ethics oversight	Before scanning, all subjects practiced the tasks. All study procedures were approved by the local Ethics Committee of UESTC. All subjects provided written consent to participate in this study. Part of the consent included the exact information about the scanning procedure and psychological assessment. The study was approved by the Ethics Committee of the clinical hospital of Chengdu Brain Science Institute (CBSI) and adhered to the latest revision of the Declaration of Helsinki

Note that full information on the approval of the study protocol must also be provided in the manuscript.

Field-specific reporting

Please select the one below that is the best fit for your research. If you are not sure, read the appropriate sections before making your selection.

☒ Life sciences ☐ Behavioural & social sciences ☐ Ecological, evolutionary & environmental sciences

For a reference copy of the document with all sections, see nature.com/documents/nr-reporting-summary-flat.pdf

Life sciences study design

All studies must disclose on these points even when the disclosure is negative.

Sample size	We did not use any statistical analysis to determine the sample size, however, since this is a within group fMRI study, we made sure the sample size was more than n>15 which is recommended by (Juha Pajula and Jussi Tohka ,2016 , doi: 10.1155/2016/2094601)
Data exclusions	To exclude subjects with high levels of depression or anxiety at pathological-relevant levels, participants were screened by means of two scales (Chinese Beck Depression Inventory, C-BDI 49; State-Trait Anxiety Inventory, STAI).
Replication	To be able to replicate results, we scanned each subject for two sessions. We then examined the effect of the mask in each session to ascertain the variability and/or consistency of our results. Also, during the analysis we conducted a check on the resting state data of task-positive and negative regions of interest, this was done partly to observe patterns similar to other resting state result especially in the default mode networks. Also, upon our re-analysis, all results remained unchanged
Randomization	The experiment involves two significant parts;mask-on and mask-off, which was randomized for pre-scan and post-scan. In this step re randomized the wearing of mask before and after the scan for all subjects in ABBA manner
Blinding	We did not use blind method for group allocation for investigator as this study involved only one group and experiment we included did not require such.

Reporting for specific materials, systems and methods

We require information from authors about some types of materials, experimental systems and methods used in many studies. Here, indicate whether each material, system or method listed is relevant to your study. If you are not sure if a list item applies to your research, read the appropriate section before selecting a response.

Materials & experimental systems

n/a	Involved in the study
☒	☐ Antibodies
☒	☐ Eukaryotic cell lines
☒	☐ Palaeontology and archaeology
☒	☐ Animals and other organisms
☒	☐ Clinical data
☒	☐ Dual use research of concern

Methods

n/a	Involved in the study
☒	☐ ChIP-seq
☒	☐ Flow cytometry
☐	☒ MRI-based neuroimaging

Magnetic resonance imaging

Experimental design

Design type

We employed both resting-state and task-based experiment. The task was block designed

Design specifications

There were three separate experiment in this study with each having specific design as follows;

Finger tapping task:When the task started, a fixation was presented and lasted 34s, then an instruction that was also the task sentence ('please move your finger') was presented and lasted 24s, and this pattern was repeated. Except for the first fixation, the rest of the fixations, as well as instruction, lasted 24s. The total task time was 226s.

Emotional face matching task: the task consisted of 2 runs and each run comprised 6 blocks of facial stimuli as well as 2 blocks of non-facial stimuli. We utilized the Asian facial stimuli obtained from the Asian facial expression database 65. During the face-processing blocks, a trio of condition-specific (happy, angry, or neutral expressions) facial stimuli were presented on the screen. Each block comprised 4 condition-specific trials, balanced for gender. During the non-facial blocks, a trio of simple geometric shapes (circles and ellipses) was presented on the screen. Similarly, participants were required to select one of the two shapes (bottom) that matched the target shape (top). All blocks started with a brief instruction (“face match” or “shapes match”) that lasted 2 seconds. Within each block, each trial was presented for 4 seconds with an interstimulus interval (ISI) of 1-3s (mean, 2s). The total task time was 336s.

Working memory task: Participants performed an n-back task 66 consisting of letters which required the maintenance and continual update of relevant working memory information. The n-back task had two different levels of complexity: 1-back and 2-back task aimed at sustaining loads and mental manipulation. Participants also performed a control task (0-back) in which they were asked to identify a prespecified letter (i.e., an “X”). During 1-back task, participants were required to identify whether the letter appeared on the screen (the “target” stimulus) matched the letter previously presented on the screen (the “cue” stimulus). Similarly, during 2-back task, participants were asked to compare whether the letter that appeared on the screen (the “target” stimulus) matched the one before the previously presented letter on the screen (the “cue” stimulus). Participants were asked to respond by pressing buttons 2 or 3 if the target was identical or different from the cue, respectively. In order to reduce visual and phonological strategies, we used phonologically closed letters with upper and lower case. Thus, the following characters were presented: b, B, d, D, g, G, p, P, t, T, v, V. Participants were told to ignore the case of the letters. Letters were presented for 500ms with a fixed interstimulus interval of 1500ms. There were 4 runs, each run had 3 blocks (0-1-or 2 block), each block had 36 trials including 12 targets. Prior to each task block, an instruction screen (0, 1-, or 2-back) was presented for 2000ms. A 4000ms blank screen separated the instructions from the onset of the first letter. Task blocks were separated by 8000ms fixation cross. The total task time was 270s.

Behavioral performance measures

We recorded the accuracy and response time for all experiments through and button press. Mean scores of accuracy and response time were computed for further analysis

Acquisition

Imaging type(s)

We obtained both functional and structural T1w images

Field strength

3T

Sequence & imaging parameters

TR/TE = 2000ms/30 ms; field of view = 240×240mm2; flip angle = 90°; matrix size = 64 × 64 and thickness = 4 mm. Axial slice number = 42 with slice thickness = 3mm and gap=0

Area of acquisition

We obtained whole-brain scans of each participant

Diffusion MRI ☐ Used ☐ Not used

Preprocessing

Preprocessing software

DPARSF1 (http://rfmri.org/DPARSF). Release = V5.0_200401
Segmentation using Dartel, and smoothed with kernel size of 8 x 8 x8, scubbing was applied with framewise displacement thresholded at 0.5 (FD_power)

Normalization	The normalization was done by Dartel (non-linear registration method) using unified segmentation on T1 image, through three steps process; co-registration, segmentation and writing normalization parameters
Normalization template	There is no “standard template”. However, DARTEL created group specific template which was applied applied to all functional images, and thus normalize into MNI space
Noise and artifact removal	We did not regress global signal . The head movement parameters were estimated in the three directions (x, y, z) along with the angular rotation on each axis (pitch, roll, and yaw) using the Friston 24-parameter model . We set the motion threshold at translation < 2mm and rotation < 2° to avoid extreme head motion, subjects with values more than the thresholds were excluded in the further analysis.
Volume censoring	scrubbing and spike regression was applied to the data, we used the algorithm implemented in the DPARSF1 (http://rfmri.org/DPARSF). Release = V5.0_200401

Statistical modeling & inference

Model type and settings	Statistical analysis of fMRI data using a mass-univariate approach based on General Linear Models (GLMs).<br><br>We used the GLM design matrix, fMRI data files and filtering, estimation of GLM parameters and inspection of results to produce Statistical Maps . Specifically, in the first level analysis (within-subject)we used random effect analysis in contrast images (computed using one sample t-test)obtained from each participant to make statistical inference for each mask_wearing condition. In the second level, random effect was used on the summery statistics of the first level, we then computed a paired t-test based on each mask condition.
Effect(s) tested	The main effect test in the study was the neural correlate of mask (oxygen saturation) before and after scanning, to test this we used factorial design to examine the overall effects.

Specify type of analysis: ☐ Whole brain ☐ ROI-based ☒ Both

Anatomical location(s)	In both the whole-brain and ROI analysis we used the Anatomical Automatic Labeling (AAL) as our reference for landmarks on the brain, specifically the ALL 116 template was employed.
Statistic type for inference (See Eklund et al. 2016)	Cluster-wise statistics was used.<br>Parameter involved: within-subject paired t-test
Correction	We applied FDR implemented in the SPM12 toolbox (https://www.fil.ion.ucl.ac.uk/spm/software/spm12/) for out multiple comparison task analysis. Here we provide a p-value =0.05 which uses the height threshold to produce local maxima and clusters and the corresponding FWE p-values and FDR q-values for peaks and clusters.

Models & analysis

n/a	Involved in the study
☒	☐ Functional and/or effective connectivity
☒	☐ Graph analysis
☒	☐ Multivariate modeling or predictive analysis
